# Comparative analysis of plant genomes allows the definition of the "Phytolongins": a novel non-SNARE longin domain protein family

**DOI:** 10.1186/1471-2164-10-510

**Published:** 2009-11-04

**Authors:** Marco Vedovato, Valeria Rossi, Joel B Dacks, Francesco Filippini

**Affiliations:** 1 MOLBINFO unit, Department of Biology, University of Padua, viale G. Colombo, 3 - 35131 Padova, Italy; 2 Department of Cell Biology, University of Alberta, 6-30 Medical Sciences Building, Edmonton, Alberta, Canada

## Abstract

**Background:**

Subcellular trafficking is a hallmark of eukaryotic cells. Because of their pivotal role in the process, a great deal of attention has been paid to the SNARE proteins. Most R-SNAREs, or "longins", however, also possess a highly conserved, N-terminal fold. This "longin domain" is known to play multiple roles in regulating SNARE activity and targeting via interaction with other trafficking proteins. However, the diversity and complement of longins in eukaryotes is poorly understood.

**Results:**

Our comparative genome survey identified a novel family of longin-related proteins, dubbed the "Phytolongins" because they are specific to land plants. Phytolongins share with longins the N-terminal longin domain and the C-terminal transmembrane domain; however, in the central region, the SNARE motif is replaced by a novel region. Phylogenetic analysis pinpoints the Phytolongins as a derivative of the plant specific VAMP72 longin sub-family and allows elucidation of Phytolongin evolution.

**Conclusion:**

"Longins" have been defined as R-SNAREs composed of both a longin domain and a SNARE motif. However, expressed gene isoforms and splice variants of longins are examples of non-SNARE motif containing longins. The discovery of Phytolongins, a family of non-SNARE longin domain proteins, together with recent evidence on the conservation of the longin-like fold in proteins involved in both vesicle fusion (e.g. the Trs20 tether) and vesicle formation (e.g. σ and μ adaptin) highlight the importance of the longin-like domain in protein trafficking and suggest that it was one of the primordial building blocks of the eukaryotic membrane-trafficking machinery.

## Background

Membrane-trafficking is a crucial process in eukaryotic cells. In recent years, the combination of structural biology, molecular cell biology and bio-informatics has allowed the definition of many of the key proteins families involved. Genome-wide analyses of both animals and plants, known to possess complex and tightly regulated protein-trafficking systems, have shown extensive sets of such membrane-trafficking protein machinery [1,2]. Among these, the soluble NSF attachment protein receptors (SNAREs) play a central role in the control of membrane fusion and of protein and lipid traffic [3,4]. SNAREs have been divided into major groups based on either their presence in the vesicle (v-SNAREs) or target membrane (t-SNAREs) or based on the presence of a conserved critical residue in the 0 polar layer, either arginine (R-SNAREs) or glutamine (Q-SNAREs) [5].

Despite being best characterised in animals, plants and fungi, SNAREs are, in fact, conserved features of the eukaryotic membrane-trafficking system. Comparative genomics and molecular phylogenetics have shown that the four major SNARE super-families (see [6] for a recent update on SNAREs classification) were already present in the Last Common Eukaryotic Ancestor (LCEA) [7]. The syntaxins or Qa-SNARE super-family has been examined in detail, demonstrating that even the five major organelle and pathway specific families had already evolved before the emergence of the current eukaryotic super-groups [8,9].

The cytoplasmic region of some R-SNAREs, the short VAMPs or "Brevins" (e.g. animal synaptobrevins, yeast Snc1/2), consists of simply the SNARE motif. However, many R-SNAREs also possess a conserved amino-terminal Longin Domain (LD), thus characterizing a large family of long VAMPs or "Longins" [10]. The longins are divided in three main families based on homology to prototypical proteins Ykt6p, Sec22b and TI-VAMP/VAMP7; the LD of Ykt6 and Sec22b show the same globular fold, based on a five-stranded β-sheet core sandwiched by one α-helix on one side and two α-helices on the other [11]. The LD of Ykt6p contains a hydrophobic patch that can inhibit the formation of a fusion complex by intramolecular binding to the coiled-coil domain (SNARE motif); mutation of a conserved Phe residue within this patch abrogates this interaction [12]. Recently, residues in the SNARE motif that are crucial to bind the LD have been identified for Sec22b [13]. Many of these residues are conserved and the same as those involved in SNARE motif binding in TI-VAMP/VAMP7 [14]. Intriguingly, the LD of human TI-VAMP/VAMP7 is capable of playing a dual role because, in addition to negatively regulating the ability of either TI-VAMP/VAMP7 or a LD-synaptobrevin chimera to participate in SNARE complexes, it is also able to target TI-VAMP/VAMP7 to the late endosomal compartment by interacting with the δ subunit of the AP3 adaptor complex [15] and to interact with the ArfGAP HRB in retrieval from the plasma membrane [14,16]. Such capacity to regulate subcellular localization (SCL) is shown also by the LD of the *Arabidopsis thaliana *VAMP7 proteins [17] and of mammalian Ykt6 [18,19]. In mammals, the LD seems also to play a relevant role in regulating neuronal development, as it is crucial to the control of neurite outgrowth [20-22].

Several lines of evidence suggest LD proteins play a central role in trafficking. Firstly, longins are the prototypical R-SNAREs and are essential in eukaryotes, whereas brevins are limited to opisthokonts and synaptobrevins are even more limited taxonomically [23]. Secondly, the LD *sensu stricto *can also be present in non-SNARE proteins: e.g. mammals have - in addition to the SNARE longin Sec22b - two homologous proteins, Sec22a and Sec22c, which lack the SNARE portion but are involved in early secretory trafficking [24]. As well, alternative splicing of the SYBL1 gene results in encoding the SNARE longin TI-VAMP/VAMP7 and two isoforms showing reverse domain architecture: isoform ''c'' (with the regular SNARE motif but missing the LD [15]) and isoform ''b'' (with the regular LD but missing the SNARE motif). Finally, the longin-like fold is not limited to members of the SNARE proteins family but rather is shared by other important trafficking protein families, such as the σ and μ subunits in clathrin adaptor complexes [25], the SEDL/Trs20p subunit of the TRAPP complex [26,27], the SRX domain of the Srα subunit of the signal recognition particle (SRP) [28,29], as well as the CHiPS and DUF254 proteins [30]. Very recently, the syndecan-binding protein synbindin, involved in neuronal membrane trafficking, has been found to show a ''special'' LD-like fold, structurally related to SEDL and split by a loop insertion corresponding to an atypical PDZ domain [31].

Although the three longin families (Ykt6, VAMP7 and Sec22) have been identified in comparative genomic analyses of SNARE proteins from many eukaryotes [23], their evolution and diversity has not been fully explored. It is thus not entirely clear whether or not they represent robust clades that branched before the extant eukaryotic supergroups and whether there are any, as-yet, unreported longin families. In order to analyze the complement of LD proteins both in number and genomic structure, we have undertaken a thorough bioinformatic analysis of publicly available completed genomes from diverse eukaryotes, with special emphasis on plant genomes, from both land plants and algae. Trafficking in plants is not only involved in canonical cellular processes but also in regulation of cytokinesis, gravitropism, responses to pathogens and abiotic stress [32]. As such, plants provide an important handle for shedding light on the pivotal role of trafficking in regulating (and mediating) cell function and differentiation.

Here, the three major longin families are demonstrated to be robustly monophyletic and to each contain the diversity of eukaryotes, thus confirming that the gene duplications giving rise to these families pre-date the LCEA [6]. In addition to the known longin families, however, our analysis has allowed the definition of a novel, plant-specific, LD protein family, the Phytolongins. We here characterise this family *in silico *in terms of genomic complement and structure, protein domain architecture and topology and structural modeling: this shows that a well-conserved N-terminal LD is present in members of this family, as is a predicted C-terminal trans-membrane region. Moreover, the unique central region of Phytolongins - showing neither detectable homology to the SNARE motif nor conservation of hydrophobic heptad repeats - is putatively able to intramolecularly bind the longin domain through a short, SNARE-like motif. Phylogenetic analysis pin-points the Phytolongins as a derivative of the plant specific VAMP72 longin family and allows elucidation of Phytolongin family evolution.

## Results and discussion

### Comparative genomics identifies unusual longin proteins

In order to address the evolution and diversity of longins and LD proteins in eukaryotes, we scanned available completed genomes from across eukaryotic diversity. Our sampling was intentionally broad and shallow in most lineages in order to obtain a tractable dataset of LD family proteins for analysis. This sampling included at least one representative of each of the five eukaryotic supergroups [33] for which genome sequences are publicly available. However, we sampled the Plant lineage in considerable depth. This included representatives of dicots (*Arabidopsis thaliana *[34], and *Populus trichocarpa *[35]), monocots (*Oryza sativa *[36]), moss (*Physcomitrella patens *[37]), as well as the multicellular chlorophyte alga *Volvox carteri*(http://www.jgi.doe.gov/Volvox, 2007) and single-celled chlorophyte and prasinophyte algae (*Chlamydomonas reinhardtii *[38] and *Ostreococcus tauri *[39]).

Genomes, transcriptomes and corresponding inferred proteomes of such organisms were scanned by iterative homology searching. Originally, we used the sequences of all known longin proteins from *Arabidopsis thaliana *as probes to scan genomes/transcriptomes/proteomes of the organisms listed above. Homologous extracted hits were in turn used as probes for iterative scanning steps: this process stopped when the search resulted in extracting no further homologous sequences. As a next step, all non-Arabidopsis candidate homologues were used as blast query sequences to be compared to *Arabidopsis thaliana *longins in order to group them based on classification of the main longin subfamilies (Ykt6, Sec22b and VAMP7) [11] and further division of plant VAMP7 proteins in two classes: VAMP71 and VAMP72 [40]. In accordance with previous studies, homologues of the three major LD family proteins were identified from the vast majority of eukaryotic genomes (Additional file 1).

The distribution and organization of the "classic" plant longins is presented in Additional file 2. Similar to animals, algae genomes have single Ykt6 and Sec22b genes. However, duplication of Ykt6 is conserved in all land plants, which also show two to four Sec22b-like genes. In plants, which indeed lack orthologues of animal brevins [23], a progressive amplification of the VAMP7 longin subfamily is observed [40]. We found that - in all scanned complete genomes - the VAMP72 complement is larger than VAMP71; moreover, the single VAMP7 gene of *Ostreococcus tauri *belongs to the VAMP72 group (Additional file 2). In general, land plants show a 2-4 fold amplification of the complement of classical longins with respect to algae: 12-18 (*Physcomitrella patens*, *Populus trichocarpa*) vs. 3-7 (*Ostreococcus tauri*, *Chlamydomonas reinhardtii*) genes. This detailed examination of the longin superfamily organisation emphasizes the increased trafficking complexity that has accompanied the colonization of land by the streptophytes and also allowed us to identify several unusual plant longin proteins.

### VAMP727 possesses a unique acidic loop in its longin domain

**Figure 1:**
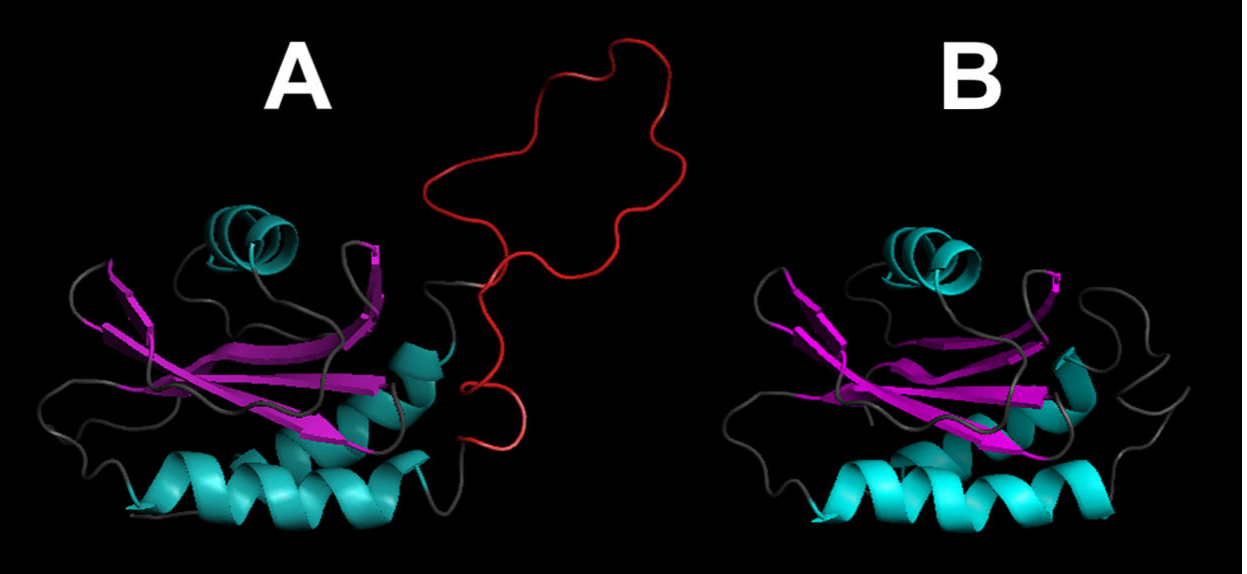
**Models of the LDs of *Arabidopsis thaliana *VAMP727 [UniProt: Q9M376] (panel A) and of its homologue VAMP725 [UniProt: O48850] (panel B), obtained using the NMR structure of the LD from human TI-VAMP/VAMP7 **[PDB: 2DMW]** as a template**. The acidic loop of VAMP727 is coloured in red. Homology modeling was performed using Geno-3D [42]; cartoon representations were obtained using PyMOL.

Since *Arabidopsis thaliana *VAMP727 [UniProt: Q9M376] shows an insertion of several amino acids in the LD sequence, which is unique amongst VAMP7 proteins [41], we performed a comparative sequence and structural analysis of this region in plant longins. Modeling of the LDs of *Arabidopsis thaliana *VAMP727 and of its closest homologue VAMP725 [UniProt: O48850] shows that the insertion sequence corresponds to an acidic extension of the loop between helices α-2 and α-3 of the LD (Figure 1). Intriguingly, this loop in the LD of Sec22b is part of a conserved interaction surface involved in binding to Sec24 within the Sec23/24/22b complex and in binding and packaging Sec22b by COPII [PDB: 2nut] [13]. When considering that such LD-complex binding is crucial to subcellular targeting, the acidic loop is likely to mediate/regulate the specific SCL of VAMP727 by steric hindrance and/or polar/charge interactions. VAMP727 are present only in seed plants (Spermatophyta) [41]. In more ancient divisions of streptophytes (e.g. Coniferophyta, Gnetophyta) the polar loop is already apparent; however, it is shorter and less acidic than in flowering plants (Magnoliophyta). It is particularly well conserved in Magnoliids, Monocotyledons and Eudicotyledons (Additional file 3).

### Plants possess non-SNARE longin proteins

A few non-SNARE LD proteins have been reported, including mammalian Sec22 gene isoforms Sec22a and Sec22c [11,24]; we report here that plants also have non-SNARE Sec22 genes. A Sec22-like rice protein [UniProt: Q6UU98] - confirmed by FLcDNA [GenBank: AK240832] and by ESTs [GenBank: AK240832, CB632349 and AU057789] - shows a complete LD sequence but lacks both the SNARE motif and the C-terminal TMD. When comparing the transcript to the corresponding genomic sequence (Chromosome 8), it is clear that this results from genomic deletion of the region encoding the SNARE motif in Sec22 paralogues. Although the exon encoding the TMD is conserved, this domain is lost because of a frame shift resulting from the new exon-intron boundary. Hence this Sec22-like protein from rice is expected to correspond to a longin domain, with no further regions. This is not surprising, when considering that single-domain proteins based on the longin fold (e.g. σ adaptin, SEDL) are known to play important roles in trafficking multi-subunit complexes.

### Identification and primary structure of the Phytolongins

Overall, our comparative genomic survey identified several unusual aspects of longin proteins in plants. However most surprisingly, in addition to members of the three well-known longin families, land plant genomes encode a family of previously unreported LD proteins which - based on *in silico *characterization (see below) - were named "Phytolongins". A first set of Phytolongins was originally identified using VAMP7 sequences from each species as sequence probes. Extracted hits, used as probes in iterated search cycles, allowed for the identification of further homologous sequences. Phytolongins share, with all longins, the N-terminal LD sequence and, with VAMP7-like and Sec22b-like longins, the C-terminus. Topology and TMD predictions (see methods), as well as presence of highly conserved residues in the C-terminus identify a putative TMD, suggesting that most probably Phytolongins are integral membrane proteins sharing topology with longins.

**Figure 2:**
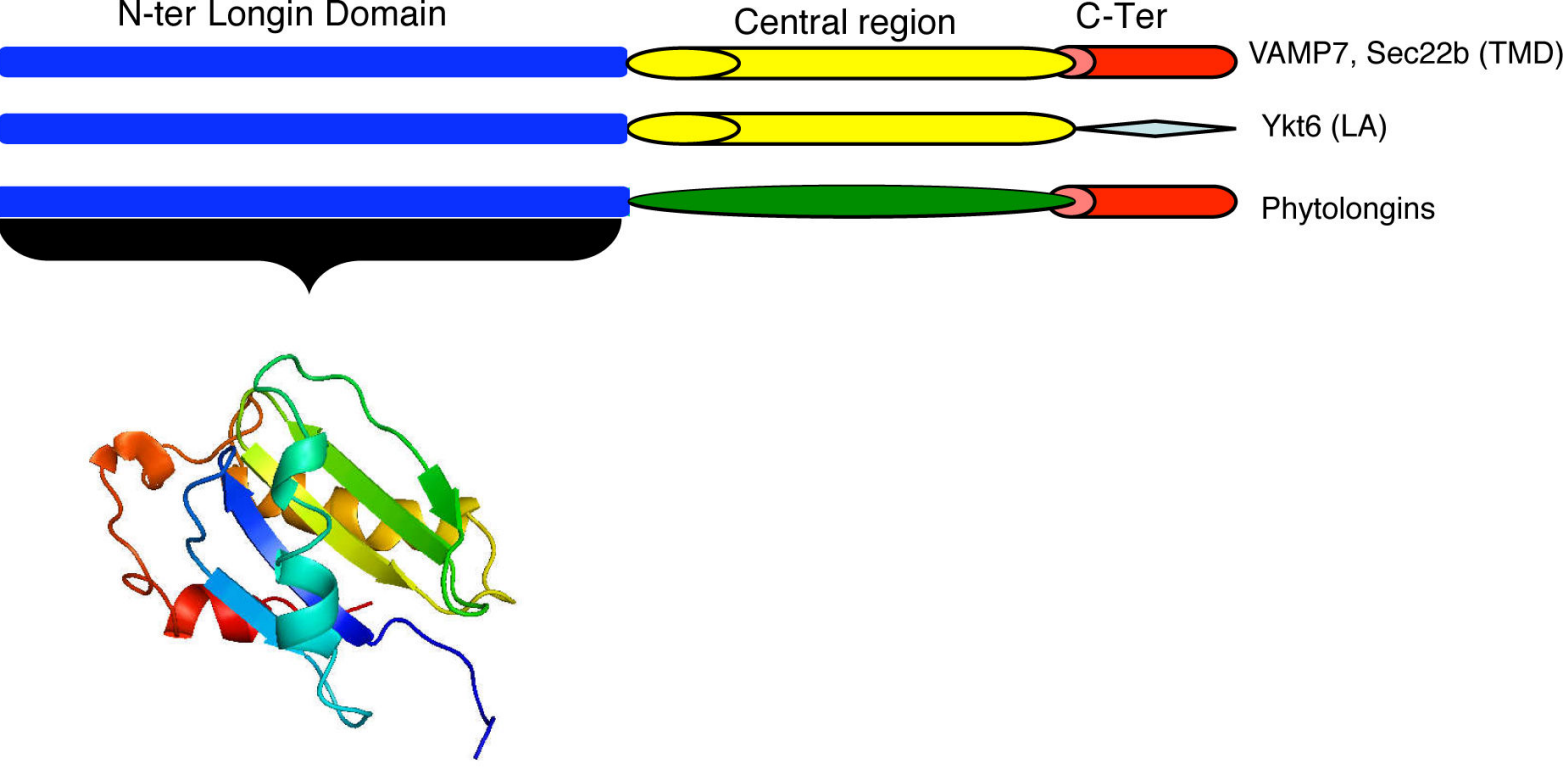
**Domain architecture of longin proteins**. This figure illustrates the common structural elements of longin proteins, including the novel Phytolongins. The central region may be a SNARE motif (yellow) in the longins Ykt6, Sec22 or VAMP7 or a PhyL region in the Phytolongins. Beneath the N-ter (longin) region is a prediction of the tertiary structure of the domain. Note that Ykt6 does not have a CTD region, with a lipid attachment (diamond) while the others possess a transmembrane domain (TMD-red cylinder).

However, the R-SNARE motif of longins is replaced in all Phytolongins by a central region (PhyL region) of unknown function consisting of roughly 60-90 amino acids (Figure 2). When using whole Phytolongin sequences or sequence fragments corresponding to their PhyL regions as probes to scan non-redundant protein or DNA sequence databases, no similarity to either SNARE motifs or any other domain was found. Further attempts, performed optimizing BLAST parameters in order to extract weakly similar sequences, confirmed that PhyL sequences are unique and specific to Phytolongins. Moreover, all homology searches confirmed the absence of Phytolongin orthologues in organisms other than land plants.

**Figure 3:**
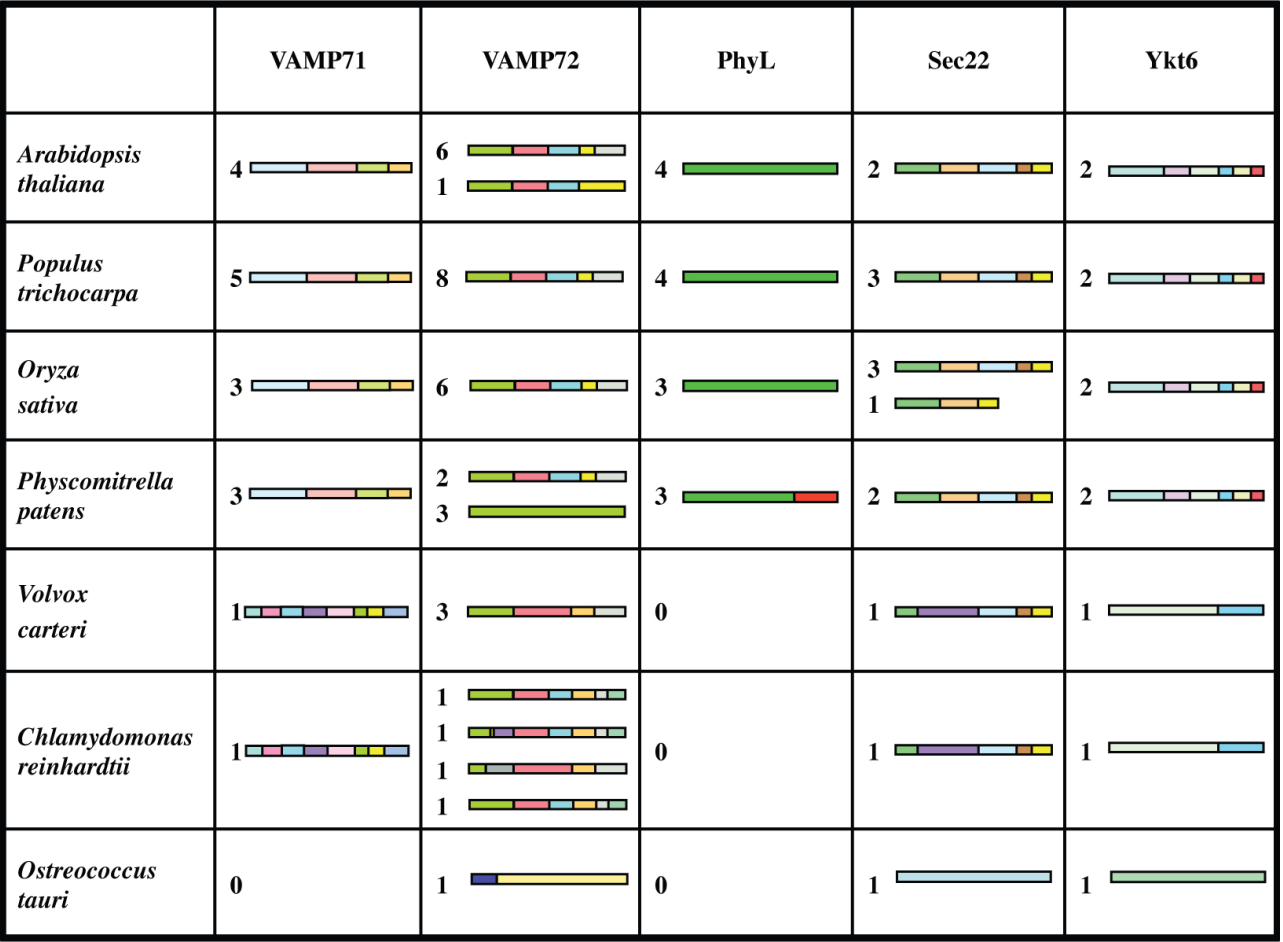
**Complements and genomic structure of plant longins**. Whole bars correspond to protein coding regions only. Bar fragments with different colours correspond to protein sequence regions encoded by different exons. Complement (numbers of members) for each longin subfamily is reported at the left side of each bar.

In order to assess the conservation of genomic organisation of the plant longins, comparison of genomic structures (i.e. exon-intron splitting of paralogues and orthologues) was performed, with the verified genomic structure of each longin gene from the scanned complete plant genomes determined by comparing genomic vs. cDNA sequence. Figure 3 illustrates conservation and variation of gene splitting patterns in plant longins. Color-coding in the figure emphasizes that some exon patterns between land plants and algae are better conserved in some longin subfamilies than in others. For example, in land plants, a four-exon pattern is fully conserved in all VAMP71 genes (i.e. in both paralogues and orthologues), whereas the single VAMP71 genes from algae show a different eight-exon pattern and do not share exon-intron junctions with land plant orthologues. Similarly, all Ykt6 genes from land plants share the same six-exon pattern, which is quite different from the mono/bi-exonic pattern of algae genes. Sec22 genes from land plants show a conserved gene-splitting organization (except for the non-SNARE Sec22 gene described above); however, the three-exon organization of their 3' halves (roughly encoding SNARE motif and TMD) is conserved also in algae. The picture of VAMP72 gene organisation is more complex: most land plant genes show a five-exon division of the coding sequences, but three VAMP72 genes are monoexonic in moss and one of the *Arabidopsis thaliana *VAMP72 genes shows merging of the last two exons (yellow and grey in figure 3). Comparison with algal VAMP72 genes shows conservation of some splitting points: for instance, division between first (light green) and second (pale red) exon. Deeper sequence comparison confirms conservation also in splice junction sequence boundaries. Two of the three longins of *Ostreococcus tauri *are monoexonic, and the third is biexonic. Finally, the Phytolongin genes are monoexonic in both dicots and monocots (this was confirmed by extending the analysis to Phytolongins from further species as well), whereas moss Phytolongins are biexonic. Overall this analysis confirmed transcription of several, but not all, predicted genes and identified novel, unreported gene structures. It also confirmed expression of Phytolongins from four plant taxa, validating the predicted genes.

### Domain architecture of the Phytolongins

Since the profile for the LD [PROSITE: PS50859] was detected in several, but not all Phytolongin sequences, structural modeling of both profile-positive and profile-negative Phytolongins was performed.

**Figure 4:**
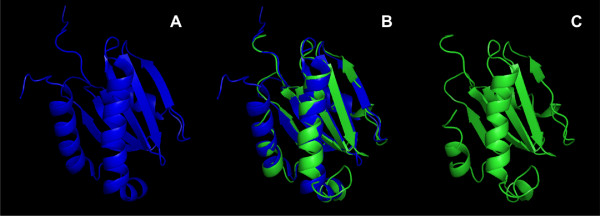
**Structural model for the LD of a Phytolongin**. The putative structure (A, blue) of the LD from an *Arabidopsis thaliana *Phytolongin [UniProt: Q9SN26] is superimposed (B) to the NMR template structure (C, green) of the LD from human TI-VAMP/VAMP7 [PDB: 2DMW]. Homology modeling was performed using Geno-3D [42]; individual cartoon and superimposition representations were obtained using PyMOL.

Figure 4 shows a model of the putative LD of a representative *Arabidopsis thaliana *Phytolongin [UniProt: Q9SN26]. Homology modeling was performed using Geno3D [42]; as a template, the NMR structure of human TI-VAMP/VAMP7 LD [PDB: 2dmw] was found to be better than LD structures from either Sec22b [PDB: 1ifq] or Ykt6p [PDB: 1h8m]. Intriguingly, structural variation was found in the α1 side of the LD, which is involved in intramolecular binding to the SNARE motif in both Ykt6p [12] and Sec22b [13].

**Figure 5:**
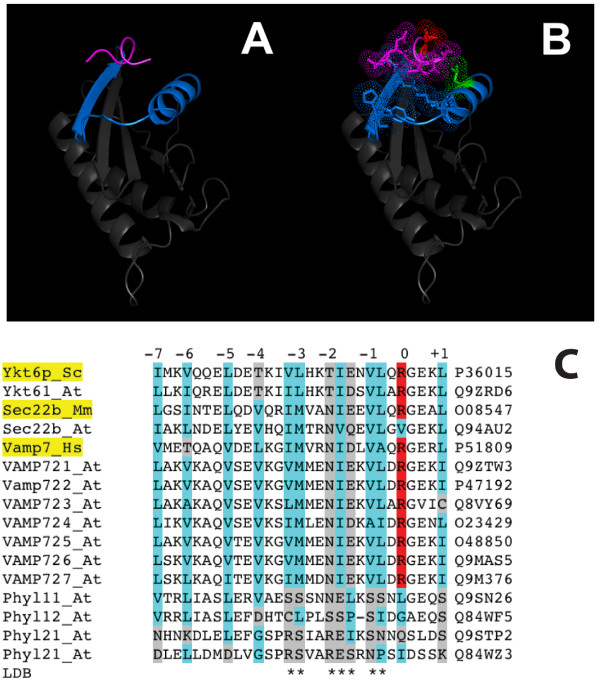
**Phyre (threading method) based prediction of intramolecular binding in a representative Phytolongin [UniProt: Q9SN26]**. Panel A: a short motif from the PhyL region (magenta) is suggested to bind to the α1-β3 region (blue) of the LD (grey). Panel B: binding is likely based on polar side chains (LD, blue; PhyL motif, magenta); hydrophobic side chains from the LD are green and the only one from the PhyL region is red. Structural representations were obtained using PyMOL. The alignment in panel C is centered around the LD binding (LDB) motif of structurally solved longins (highlighted in yellow). Homologous Ykt6, Sec22b and VAMP7 family longins and the four Phytolongins from *Arabidopsis thaliana *are also included. The conserved Arg residue at the zero layer of the SNARE motif is highlighted in red. Hydrophobic or polar residues are highlighted in respectively cyan and grey in columns concerning the heptad repeat layers or the LDB. The putative LDB region of Phytolongins is clearly more polar than LDB of longins, and the Arg residue is not conserved. Instead, several hydrophobic layer positions are conserved with the Phytolongins. Conservation is not apparent in the CT half (not shown).

In order to obtain a model including both the LD and PhyL regions, whole Phytolongins were used as sequence probes in fold recognition based modeling. Phyre [43,44] confirmed that the LD of TI-VAMP/VAMP7 LD is the best available template for a Phytolongin LD; in addition however, it was also able to propose a model superimposed onto the structure of subunit Sec22b of the COPII complex recently solved [PDB: 2nup, chain c] [13]. In particular, the model in figure 5a shows that a short peptide from the PhyL region (magenta) is close to the α1-β3 region (blue) of the LD, i.e. to the SNARE-binding site [12,13].

Threading predictions were iterated and the presence of the putative LD binding motif was confirmed for the PhyL regions of all Phytolongins (data not shown). When considering that the α1-β3 region is also a binding partner for the SNARE-like region of Hrb [14], it is not surprising to see that the putative LD-binding peptides of the PhyL regions are aligned in the model to the LD binding motif of the template and that the putative interaction is based on polar rather than hydrophobic interactions (Figure 5, panels b and c). Figure 5c also shows that the NT half of the PhyL region, including its putative LD binding motif, shares with SNARE motifs some heptadic, hydrophobic layers (whereas the CT half does not - data not shown). Absence of overall homology to the SNARE motif, presence of a putative LD-binding motif and conservation of the heptadic layers only in the NT half suggest that the PhyL region might share with the SNARE motif capacity to bind the LD, but not to participate in SNARE bundles, thus resembling the SNARE-like region of Hrb [14].

The PhyL region is likely to have strongly diverged from the SNARE motif by point mutations and/or sequence insertions. High divergence between the PhyL region and SNARE motif, together with α1 sequence divergence between Phytolongins and longins LDs suggest that different longin domain proteins may show different binding properties. Indeed, even among SNARE longins from the same organism - e.g. yeast - the intramolecular binding mechanism can be either clearly apparent (Ykt6p [12]) or not detected (Nyv1p [45]). Putative binding of the PhyL region to the LD is in agreement with evidence that non-SNARE proteins can also bind the LD [14,15].

In order to obtain further functional predictions, PhyL region sequences from all identified Phytolongins were scanned for the presence of PROSITE motifs/signatures (see methods for details). When searching for degenerate patterns, putative calcium binding regions were consistently found (data not shown) but no positional conservation of these putative sites in multiple alignment was observed. While false positives among degenerate versions of low complexity motifs are quite common, this low confidence prediction is reported because of the special significance of calcium binding in trafficking proteins [46].

Overall, the domain modeling shows that, despite no detectable sequence homology with SNARE motifs, Phytolongins are *bona fide *longin proteins with conserved longin domain structure and a potentially conserved binding mechanism between the LD and PhyL motif.

### Evolution of the Phytolongins

**Figure 6:**
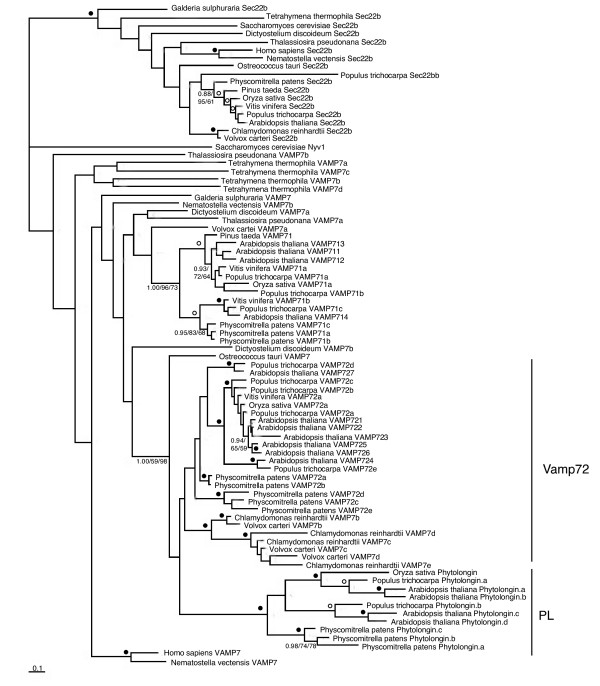
**Phylogeny of Sec22b, VAMP7 and Phytolongins**. This figure demonstrates that Phytolongins are most likely derived from within the VAMP72 clade of plants. The Phytolongins (PL) and Sec22b clades are denoted by vertical bars. In this, and all subsequent phylogenetic figures, the best Bayesian topology is shown, with support values for resolved nodes in the order of posterior probabilities, PhyML bootstrap values and RAxML bootstrap values. Values are not provided for nodes supported by less than 0.80 posterior probability and 50% bootstrap support by both methods. For some well-supported nodes, values are replaced by symbols with closed circles denoting better than 1.00/95/95 and open circles denoting better than 0.95/80/80 support.

Having established that the Phytolongins are LD proteins, we wanted to establish the longin family from which they are derived. A variety of datasets were created to address this question and were analysed using Bayesian and two methods of protein maximum-likelihood phylogeny. Initial analyses of longins from diverse eukaryotes clearly resolve the Phytolongins as a monophyletic group to the exclusion of all other sequences. The overall analysis (Additional file 4) did not resolve the placement of this clade but did resolve the Ykt6 sequences as monophyletic (0.99/92/90 posterior probabilities/PhyML/RAxML bootstrap support, respectively) indicating that the Phytolongins are not derived from within this family. Subsequent analysis further excluded the Sec22 family as a source of the Phytolongins, with a strongly supported node resolving the Sec22 family and allowing the establishment of the Phytolongins as embedded within the plant specific VAMP72 clade (Figure 6).

**Figure 7:**
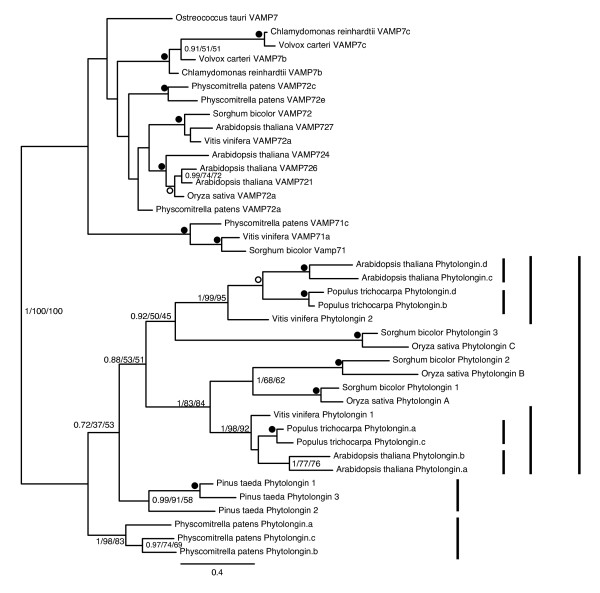
**Phylogenetic analysis of the Phytolongin family**. This figure shows the results of an analysis of Phytolongin sequences with selected plant VAMP7 homologues as outgroups. The small inner vertical bars denote the clades of *Populus trichocarpa *and *Arabidopsis thaliana *expansions. The middle vertical bars denote the well-supported rosid-specific expansions while the outer vertical bars denote the clades of angiosperm, gymnosperm and bryophyte Phytolongins respectively.

In order to further investigate the internal evolution of the Phytolongin family, a final dataset was analysed (Figure 7). Independent clades of Phytolongins were observed in the bryophytes (*Physcomitrella patens*), gymnosperms (*Pinus taeda*) and the angiosperms. Although the node separating the bryophytes from the other plant Phytolongins is poorly resolved in figure 7, subsequent analyses provided more robust support (Additional file 5-1.00/56/80). Within the angiosperms, two major clades are apparent. Although the inclusion of the monocot sequences in these clades is unclear, the nodes supporting the dicot sequences in each clade are very well supported (Figure 7).

**Figure 8:**
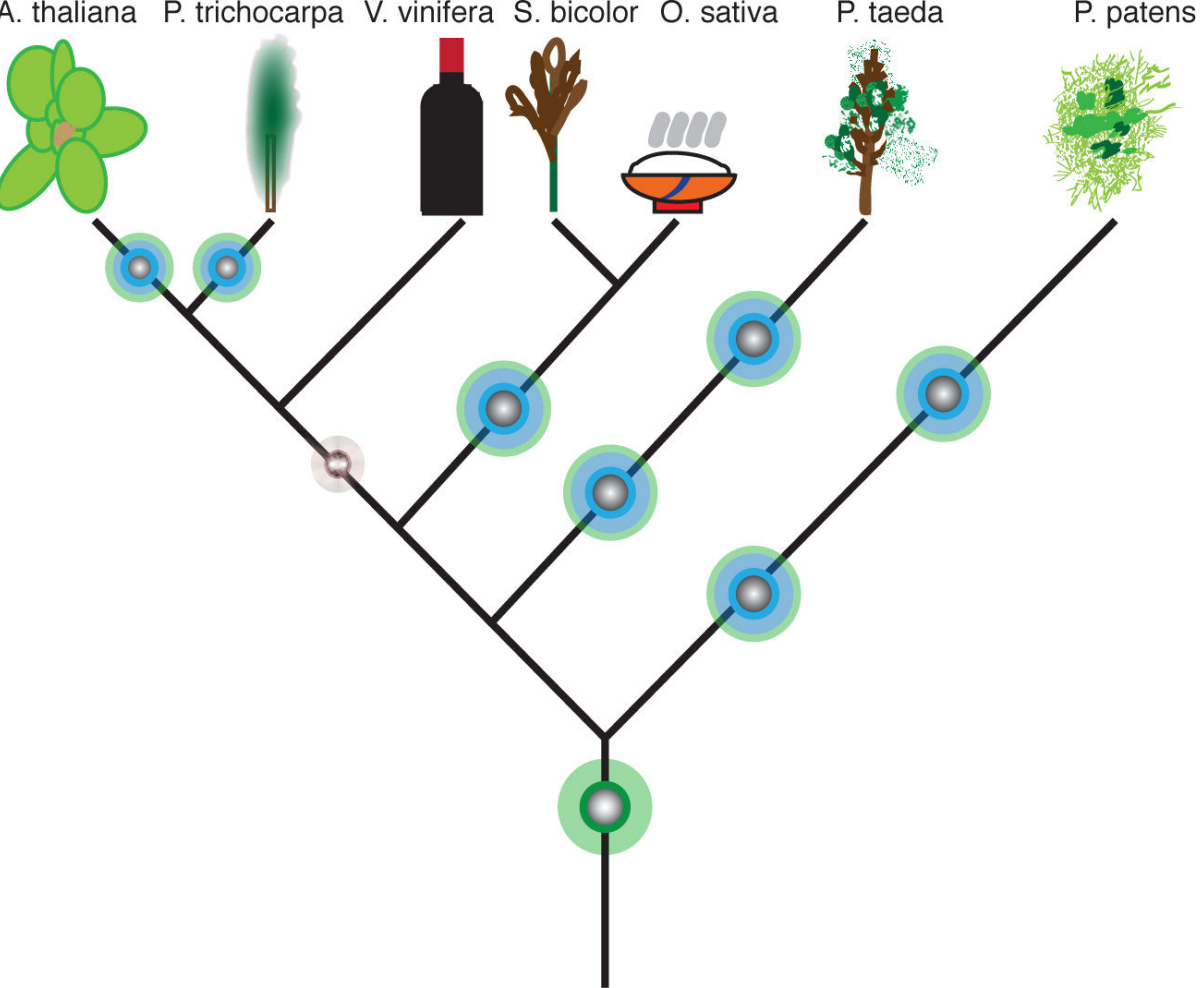
**History of the Phytolongin longin family**. This cartoon illustrates the proposed evolutionary history of the Phytolongins including the genesis of the proteins at the base of the streptophytes (green radial) and subsequent gene duplications in the various lineages giving rise to the expanded Phytolongin complements (blue radials). The brown radial prior to the separation of the monocots and dicots denotes that, although we hypothesize a gene duplication at that point, the phylogeny is not robustly supported.

Figure 8 illustrates our hypothesis of Phytolongin evolution. The ancestor of streptophytes possessed a single Phytolongin gene, as did the ancestor of tracheophytes with subsequent independent gene family expansions in the descendent lineages. It is difficult to deduce whether the duplication giving rise to the two major clades of angiosperm Phytolongins predates the separation of monocots and dicots. However, based on the observed topology, this appears to be the best-supported scenario. Nonetheless, with the two well-resolved clades of rosid Phytolongins, it is clear that the duplication had already occurred at this point (Figure 8). Further expansion of the Phytolongin gene families are also observed in the *Populus trichocarpa *and *Arabidopsis thaliana *genomes, as well as in the ancestor of *Sorghum bicolor *and *Oryza sativa*.

### Putative involvement of Phytolongins in subcellular trafficking

Preliminary data from subcellular location prediction software applied to the *Arabidopsis thaliana *Phytolongins gave results inconsistent between the different algorithms and, for the *Arabidopsis thaliana *VAMPs, results inconsistent with experimentally established location of the proteins (data not shown). Consequently this method of analysis was not pursued. Nonetheless, it is possible to speculate on the possible SCL of Phytolongins and their involvement in plant subcellular trafficking based on their similarity and derivation from the plant specific clade of VAMP72 proteins.

We performed an analysis of percent identity between the animal TI-VAMP/VAMP7, *Arabidopsis thaliana *VAMP homologues and the four Arabidopsis Phytolongins, considering (i) the full-length sequence, (ii) the LD region only and (iii) the CT region only (i.e. the SNARE motif/Phyl region + TMD). Animal VAMP7 proteins are more similar to the four VAMP71 than to the seven VAMP72 and, intriguingly, such difference is dependent on divergence at the LD sequence. In the CT region, the VAMP71 and VAMP72 share a range in similarity to the animal homologues between 38-42%, as do LDs from animal VAMP7 to plant VAMP71 LDs. However, similarity between the animal TI-VAMP/VAMP7 and VAMP72 LDs is roughly ten percent lower (31 to 34). It is therefore noteworthy that all four Phytolongins LDs are more similar to LDs from VAMP72 proteins than LDs from VAMP71. It has to be stressed here that subcellular targeting of longins is mediated by the LD [12-19], acting as a dominant signal in chimeric constructs combining domains from VAMP7 proteins with different SCL [17] Moreover, in addition to a similar LD, VAMP72 proteins and Phytolongins are likely to share a conserved intramolecular binding mechanism resulting in a closed conformation in the conformational epitope mediating subcellular targeting.

While the VAMP71 homologues are localized to the Golgi body and vacuole, all VAMP72 proteins localise to the PM/endosomal compartment [17], apart from VAMP723 (ER [17]) and VAMP727 (prevacuolar compartment [41]). Since the Phytolongins share higher similarity with the VAMP72 family, we tentatively speculate that the Phytolongins might be involved in events at the PM/endosomes as well. However, given that multiple linear and often short, cryptic motifs and conformational epitopes, as well as binding partners and post-translational modifications, can finely tune subcellular sorting, experimental evidence is expected to shed light on the SCL, interactions and role in trafficking of this novel protein family.

## Conclusion

Our bioinformatic analysis of longin proteins has both verified the ancient nature of the three R-SNARE longin subfamilies and identified the Phytolongins, a previously undescribed LD protein family, specific to plants. That Phytolongins are present in multiple plant genomes, spanning the diversity of land plants, and that Phytolongin transcripts are available from several plant EST projects speak to the validity of the predicted novel genes. The expanded nature of this gene family in many taxa speaks to its potential importance in plant biology.

In addition to this new family of non-SNARE longin proteins, we identified several splice-variants of canonical longins, missing the SNARE motif. These, together with the presence of other non-SNARE longin proteins, and the conserved longin-like fold in a variety of other trafficking proteins, all suggest that the longin domain may be a more central structural feature to membrane-trafficking in eukaryotic cells than is currently recognised. Since the longin-like fold is present in diverse trafficking machinery, involved in vesicle fusion, vesicle formation and even the signal recognition particle, we propose that the longin-like domain should join other prominent structural protein elements, such as the alpha-solenoid, and beta-propeller domains [47] and monomeric GTPases, in the list of the primordial building blocks that were involved in the earliest evolution of a eukaryotic membrane-trafficking system.

## Methods

### Genome scanning and analysis

Genome-wide searches were performed using BLAST [48] with default scoring parameters and excluding the filter for low-complexity regions. Both nucleotide, protein and translated BLAST programs were used to search for homologous genes, transcripts or proteins at both the NCBI and EBI databases as well as at the JGI genome portal http://genome.jgi-psf.org/. Searches vs. complete, non redundant NCBI and EBI databases were performed limiting organism to Eukaryota (taxid:2759); at the same time, several searches at the JGI portal were limited to specific model organisms.

Evidence regarding the conservation and variation of the intron/exon structure was obtained using available transcripts (FLcDNAs and/or ESTs) from EBI, NCBI and JGI databases as sequence queries in BLAST searches vs. genomic scaffolds. Alignment of transcript regions to genomic sequences provided a preliminary exon map of each gene. The map was then manually curated and optimized comparing corresponding translated protein fragments and taking into account splice consensus sequences.

### Protein sequence analysis and structural predictions

Scanning of canonical PROSITE motifs and signatures was performed using the ScanProsite tool [49] available at the ExPAsy server http://www.expasy.org, whereas scanning for degenerate patterns was performed using PROSITE scan available on-line at the IBCP-PBIL server http://npsa-pbil.ibcp.fr and allowing for 2 mismatches or setting for 65% similarity.

Prediction of TMD and topology was performed using PSORT [50], DAS [51], TMPRED http://www.ch.embnet.org/software/TMPRED_form.html, SOSUI [52] and HMMTOP [53].

Homology modeling and superposition of models to templates was performed using the Geno3D tool available on-line at the IBCP-PBIL server http://geno3d-pbil.ibcp.fr[42]. Fold recognition was performed using Phyre [43,44,54]. 3D representation of molecular structures was obtained using the PyMOL Molecular Graphics System http://www.pymol.org.

### Phylogenetic analysis

Sequences were aligned initially using Clustal X [55] and then adjusted manually based on known secondary structural features of the predicted longin domain. For phylogenetic analysis only regions of unambiguous homology were retained. For all datasets, details of taxon numbers, positions and models of sequence evolution are listed in Additional file 6. All alignments are available upon request and a list of abbreviations and accession numbers for all sequences used in the analyses is provided in Additional file 1.

In all analyses, the model of sequence evolution was established using the program Prot-test V. 1.3 [56]. Datasets were then processed using three methods of protein phylogenetic analysis. The optimal topology and Bayesian posterior probability values were obtained using Mr. Bayes version 3.1.2 [57] with two independent runs each of 1000000 generations. The burnin value was estimated graphically and all trees prior to the plateau were excluded from the consensus. In all cases the splits frequency was below 0.1 indicating that the two runs had converged. Protein Maximum Likelihood (ML) bootstrap support values were calculated using PHYML [58] and RAxML [59] with the appropriate models of sequence evolution and correction for variation of rates among sites. Phylogenetic analyses were performed on the CamGrid cluster at the University of Cambridge or the bioinfo cluster at the University of Alberta.

## Abbreviations used

CT: carboxy (C)-terminal; FLcDNA: full-length cDNA; LCEA: last common eukaryotic ancestor; LD: Longin domain; NT: amino (N)-terminal; PDZ: PSD95: DlgA and Zo-1; SNARE: soluble N-ethylmaleimide sensitive factor (NSF) attachment protein (SNAP) receptor; SRP: signal recognition particle; TI-VAMP: tetanus neurotoxin insensitive VAMP; TMD: transmembrane domain; TRAPP: transport protein particle; VAMP: vesicle associated membrane protein

## Authors' contributions

MV collected the dataset for the analysis, performed structural modeling and some of the phylogenetic analyses. VR participated in data mining and performed the genomic structure analyses. JBD conceived of and supervised the phylogenetic analyses, as well as performed the analyses on the Phytolongin-specific dataset. FF initiated and coordinated the work and participated in the structural modeling and sequence analyses. All authors have read and approved of the final manuscript.

## Supplementary Material

Additional file 1 **Table S1**. Accession numbers and names of all sequences identified in our comparative genomic searches and used in phylogenetic analyses. Click here for file

Additional file 2 **Table S2**. Whole complements of "classic" longins from a number of model plant species. Click here for file

Additional file 3 **Alignment of the VAMP727 loop region in diverse plants**. This figure shows a multiple alignment of the conserved VAMP727 acidic loop region including a few adjacent C-ter and N-ter residues, corresponding to regions 94-121 or 96-124 of VAMP727 proteins from respectively *Arabidopsis thaliana *or *Oryza sativa*. Loops regions are 98-116 (At) and 100-119 (Os) respectively. Click here for file

Additional file 4 **Longin phylogeny**. This figure demonstrates that the Phytolongins form a well-resolved clade within the longin family and that they are unlikely to have been derived from within the Ykt6 clade of longins. The vertical bars highlight the Ykt6 and Phytolongin (PL) clades respectively. Click here for file

Additional file 5 **Phytolongin phylogeny (altered taxon sampling)**. This figure shows the robust separation of bryophyte and gymno/angiosperm sequences. This dataset included a homologue from *Salaginella moellendorffii *but excluded a divergent sequence from Sorghum bicolor and resulted in a more robust resolution of the *Physcomitrella patens *sequences from the other plant Phytolongins (vertical bar). Click here for file

Additional file 6 **Table S3**. Characteristics of datasets used in phylogenetic analysis. Datasets are listed by name, number of taxa, number of amino acid positions, the model of sequence evolution deduced by Prot-test and the figure in the paper in which the results are illustrated. Click here for file

## References

[B1] Bock JB Matern HT Peden AA Scheller RH A genomic perspective on membrane compartment organization Nature 2001 409 839 841 10.1038/35057024 11237004

[B2] Carter CJ Bednarek SY Raikhel NV Membrane trafficking in plants: new discoveries and approaches Curr Opin Plant Biol 2004 7 701 707 10.1016/j.pbi.2004.09.016 15491919

[B3] Ungar D Hughson FM SNARE protein structure and function Annu Rev Cell Dev Biol 2003 19 493 517 10.1146/annurev.cellbio.19.110701.155609 14570579

[B4] Hong W SNAREs and traffic Biochim Biophys Acta 2005 1744 493 517 16038056

[B5] Fasshauer D Otto H Eliason WK Jahn R Brunger AT Structural changes are associated with soluble N-ethylmaleimide-sensitive fusion protein attachment protein receptor complex formation J Biol Chem 1997 272 28036 28041 10.1074/jbc.272.44.28036 9346956

[B6] Kloepper TH Kienle CN Fasshauer D An elaborate classification of SNARE proteins sheds light on the conservation of the eukaryotic endomembrane system Mol Biol Cell 2007 18 3463 3471 17596510 10.1091/mbc.E07-03-0193 PMC1951749

[B7] Dacks JB Field MC Evolution of the eukaryotic membrane-trafficking system: origin, tempo and mode J Cell Sci 2007 120 2977 2985 10.1242/jcs.013250 17715154

[B8] Dacks JB Doolittle WF Novel syntaxin gene sequences from *Giardia*, *Trypanosoma *and algae: implications for the ancient evolution of the eukaryotic endomembrane system J Cell Sci 2002 115 Pt 8 1635 1642 11950882 10.1242/jcs.115.8.1635

[B9] Dacks JB Doolittle WF Molecular and phylogenetic characterization of syntaxin genes from parasitic protozoa Mol Biochem Parasitol 2004 136 123 136 10.1016/j.molbiopara.2004.02.014 15478792

[B10] Filippini F Rossi V Galli T Budillon A D'Urso M D'Esposito M Longins: a new evolutionary conserved VAMP family sharing a novel SNARE domain Trends Biochem Sci 2001 26 407 409 10.1016/S0968-0004(01)01861-8 11440841

[B11] Rossi V Banfield DK Vacca M Dietrich LE Ungermann C D'Esposito M Galli T Filippini F Longins and their longin domains: regulated SNAREs and multifunctional SNARE regulators Trends Biochem Sci 2004 29 682 688 10.1016/j.tibs.2004.10.002 15544955

[B12] Tochio H Tsui MM Banfield DK Zhang M An autoinhibitory mechanism for nonsyntaxin SNARE proteins revealed by the structure of Ykt6p Science 2001 293 698 702 10.1126/science.1062950 11474112

[B13] Mancias JD Goldberg J The transport signal on Sec22 for packaging into COPII-coated vesicles is a conformational epitope Mol Cell 2007 26 403 414 10.1016/j.molcel.2007.03.017 17499046

[B14] Pryor PR Jackson L Gray SR Edeling MA Thompson A Sanderson CM Evans PR Owen DJ Luzio JP Molecular basis for the sorting of the SNARE VAMP7 into endocytic clathrin-coated vesicles by the ArfGAP Hrb Cell 2008 134 817 827 18775314 10.1016/j.cell.2008.07.023 PMC2648964

[B15] Martinez-Arca S Rudge R Vacca M Raposo G Camonis J Proux-Gillardeaux V Daviet L Formstecher E Hamburger A Filippini F D'Esposito M Galli T A dual mechanism controlling the localization and function of exocytic v-SNAREs Proc Natl Acad Sci USA 2003 100 9011 9016 12853575 10.1073/pnas.1431910100 PMC166429

[B16] Chaineau M Danglot L Proux-Gillardeaux V Galli T Role of HRB in clathrin-dependent endocytosis J Biol Chem 2008 283 34365 34373 10.1074/jbc.M804587200 18819912 PMC2662242

[B17] Uemura T Sato MH Takeyasu K The longin domain regulates subcellular targeting of VAMP7 in *Arabidopsis thaliana* FEBS Lett 2005 579 2842 2846 10.1016/j.febslet.2005.04.022 15876431

[B18] Hasegawa H Zinsser S Rhee Y Vik-Mo EO Davanger S Hay JC Mammalian Ykt6 is a neuronal SNARE targeted to a specialized compartment by its profilin-like amino terminal domain Mol Biol Cell 2003 14 698 720 12589064 10.1091/mbc.E02-09-0556 PMC150002

[B19] Hasegawa H Yang Z Oltedal L Davanger S Hay JC Intramolecular protein-protein and protein-lipid interactions control the conformation and subcellular targeting of neuronal Ykt6 J Cell Sci 2004 117 4495 4508 10.1242/jcs.01314 15331663

[B20] Martinez-Arca S Alberts P Zahraoui A Louvard D Galli T Role of tetanus neurotoxin insensitive vesicle-associated membrane protein (TI-VAMP) in vesicular transport mediating neurite outgrowth J Cell Biol 2000 149 889 899 10811829 10.1083/jcb.149.4.889 PMC2174569

[B21] Martinez-Arca S Coco S Mainguy G Schenk U Alberts P Bouille P Mezzina M Prochiantz A Matteoli M Louvard D Galli T A common exocytotic mechanism mediates axonal and dendritic outgrowth J Neurosci 2001 21 3830 3838 11356871 10.1523/JNEUROSCI.21-11-03830.2001 PMC6762683

[B22] Muzerelle A Alberts P Martinez-Arca S Jeannequin O Lafaye P Mazie JC Galli T Gaspar P Tetanus neurotoxin-insensitive vesicle-associated membrane protein localizes to a presynaptic membrane compartment in selected terminal subsets of the rat brain Neuroscience 2003 122 59 75 10.1016/S0306-4522(03)00567-0 14596849

[B23] Rossi V Picco R Vacca M D'Esposito M D'Urso M Galli T Filippini F VAMP subfamilies identified by specific R-SNARE motifs Biol Cell 2004 96 251 256 10.1016/j.biolcel.2003.12.009 15145528

[B24] Tang BL Low DYH Hong W Hsec22c: a homolog of yeast Sec22p and mammalian rsec22a and msec22b/ERS-24 Biochem Biophys Res Comm 1998 243 885 891 10.1006/bbrc.1998.8194 9501016

[B25] Collins BM McCoy AJ Kent HM Evans PR Owen DJ Molecular architecture and functional model of the endocytic AP2 complex Cell 2002 109 523 535 10.1016/S0092-8674(02)00735-3 12086608

[B26] Jang SB Kim Y-G Cho Y-S Su P-G Kim K-H Oh B-H Crystal structure of SEDL and its implications for a genetic disease Spondyloepiphyseal Dysplasia Tarda J Biol Chem 2002 277 49863 49869 10.1074/jbc.M207436200 12361953

[B27] Gécz J Shaw MA Bellon JR de Barros Lopes M Human wild-type SEDL protein functionally complements yeast Trs20p but some naturally occurring SEDL mutants do not Gene 2003 320 137 144 10.1016/S0378-1119(03)00819-9 14597397

[B28] Schwartz T Blobel G Structural basis for the function of the β subunit of the eukaryotic signal recognition particle receptor Cell 2003 112 793 803 10.1016/S0092-8674(03)00161-2 12654246

[B29] Schlenker O Hendricks A Sinning I Wild K The structure of the mammalian signal recognition particle (SRP) receptor as prototype for the interaction of small GTPases with Longin domains J Biol Chem 2006 281 8898 8906 10.1074/jbc.M512415200 16439358

[B30] Kinch LN Grishin NV Longin-like folds identified in CHiPS and DUF254 proteins: vesicle trafficking complexes conserved in eukaryotic evolution Protein Sci 2006 15 2669 2674 17075139 10.1110/ps.062419006 PMC2242422

[B31] Fan S Wei Z Xu H Gong W Crystal structure of human synbindin reveals two conformations of longin domain Biochem Biophys Res Commun 2009 378 338 343 10.1016/j.bbrc.2008.04.143 18466758

[B32] Lipka V Kwon C Panstruga R SNARE-ware: the role of SNARE-domain proteins in plant biology Annu Rev Cell Dev Biol 2007 23 147 174 10.1146/annurev.cellbio.23.090506.123529 17506694

[B33] Adl SM Simpson AG Farmer MA Andersen RA Anderson OR Barta JR Bowser SS Brugerolle G Fensome RA Fredericq S James TY Karpov S Kugrens P Krug J Lane CE Lewis LA Lodge J Lynn DH Mann DG McCourt RM Mendoza L Moestrup O Mozley-Standridge SE Nerad TA Shearer CA Smirnov AV Spiegel FW Taylor MF The new higher level classification of eukaryotes with emphasis on the taxonomy of protists J Eukaryot Microbiol 2005 52 399 451 10.1111/j.1550-7408.2005.00053.x 16248873

[B34] Arabidopsis Genome Initiative Analysis of the genome sequence of the flowering plant *Arabidopsis thaliana* Nature 2000 408 796 815 10.1038/35048692 11130711

[B35] Tuskan GA Difazio S Jansson S Bohlmann J Grigoriev I Hellsten U Putnam N Ralph S Rombauts S Salamov A Schein J Sterck L Aerts A Bhalerao RR Bhalerao RP Blaudez D Boerjan W Brun A Brunner A Busov V Campbell M Carlson J Chalot M Chapman J Chen GL Cooper D Coutinho PM Couturier J Covert S Cronk Q The genome of black cottonwood, *Populus trichocarpa *(Torr. & Gray) Science 2006 313 1596 1604 10.1126/science.1128691 16973872

[B36] Yu J Hu S Wang J Wong GK Li S Liu B Deng Y Dai L Zhou Y Zhang X Cao M Liu J Sun J Tang J Chen Y Huang X Lin W Ye C Tong W Cong L Geng J Han Y Li L Li W Hu G Huang X Li W Li J Liu Z Li L A draft sequence of the rice genome (*Oryza sativa *L. ssp. indica) Science 2002 296 79 92 10.1126/science.1068037 11935017

[B37] Rensing SA Lang D Zimmer AD Terry A Salamov A Shapiro H Nishiyama T Perroud PF Lindquist EA Kamisugi Y Tanahashi T Sakakibara K Fujita T Oishi K Shin-I T Kuroki Y Toyoda A Suzuki Y Hashimoto S Yamaguchi K Sugano S Kohara Y Fujiyama A Anterola A Aoki S Ashton N Barbazuk WB Barker E Bennetzen JL Blankenship R The *Physcomitrella *genome reveals evolutionary insights into the conquest of land by plants Science 2008 319 64 69 10.1126/science.1150646 18079367

[B38] Merchant SS Prochnik SE Vallon O Harris EH Karpowicz SJ Witman GB Terry A Salamov A Fritz-Laylin LK Maréchal-Drouard L Marshall WF Qu LH Nelson DR Sanderfoot AA Spalding MH Kapitonov VV Ren Q Ferris P Lindquist E Shapiro H Lucas SM Grimwood J Schmutz J Cardol P Cerutti H Chanfreau G Chen CL Cognat V Croft MT Dent R The *Chlamydomonas *genome reveals the evolution of key animal and plant functions Science 2007 318 245 250 10.1126/science.1143609 17932292 PMC2875087

[B39] Derelle E Ferraz C Rombauts S Rouzé P Worden AZ Robbens S Partensky F Degroeve S Echeynié S Cooke R Saeys Y Wuyts J Jabbari K Bowler C Panaud O Piégu B Ball SG Ral JP Bouget FY Piganeau G De Baets B Picard A Delseny M Demaille J Peer Y Van de Moreau H Genome analysis of the smallest free-living eukaryote *Ostreococcus tauri *unveils many unique features Proc Natl Acad Sci USA 2006 103 11647 11652 16868079 10.1073/pnas.0604795103 PMC1544224

[B40] Sanderfoot A Increases in the number of SNARE genes parallels the rise of multicellularity among the green plants Plant Physiol 2007 144 6 17 17369437 10.1104/pp.106.092973 PMC1913785

[B41] Ebine K Okatani Y Uemura T Goh T Shoda K Niihama M Morita MT Spitzer C Otegui MS Nakano A Ueda T A SNARE complex unique to seed plants is required for protein storage vacuole biogenesis and seed development of *Arabidopsis thaliana* Plant Cell 2008 20 3006 3021 18984676 10.1105/tpc.107.057711 PMC2613668

[B42] Combet C Jambon M Deléage G Geourjon C Geno3D: Automatic comparative molecular modelling of protein Bioinformatics 2002 18 213 214 10.1093/bioinformatics/18.1.213 11836238

[B43] Bennett-Lovsey RM Herbert AD Sternberg MJE Kelley LA Exploring the extremes of sequence/structure space with ensemble fold recognition in the program Phyre Proteins 2008 70 611 625 10.1002/prot.21688 17876813

[B44] Kelley LA Sternberg MJE Protein structure prediction on the web: a case study using the Phyre server Nature Protocols 2009 4 363 371 10.1038/nprot.2009.2 19247286

[B45] Wen W Chen L Wu H Sun X Zhang M Banfield DK Identification of the yeast R-SNARE Nyv1p as a novel longin domain-containing protein Mol Biol Cell 2006 17 4282 4299 16855025 10.1091/mbc.E06-02-0128 PMC1635351

[B46] Burgoyne RD O'Callaghan DW Hasdemir B Haynes LP Tepikin AV Neuronal Ca2+-sensor proteins: multitalented regulators of neuronal function Trends Neurosci 2004 27 203 209 10.1016/j.tins.2004.01.010 15046879

[B47] Field MC Dacks JB First and last ancestors: reconstructing evolution of the endomembrane system with ESCRTs, vesicle coat proteins, and nuclear pore complexes Curr Opin Cell Biol 2009 21 4 13 10.1016/j.ceb.2008.12.004 19201590

[B48] Altschul SF Madden TL Schäffer AA Zhang J Zhang Z Miller W Lipman DJ Gapped BLAST and PSI-BLAST: a new generation of protein database search programs Nucleic Acids Res 1997 25 3389 3402 9254694 10.1093/nar/25.17.3389 PMC146917

[B49] De Castro E Sigrist CJA Gattiker A Bulliard V Langendijk-Genevaux PS Gasteiger E Bairoch A Hulo N ScanProsite: detection of PROSITE signature matches and ProRule-associated functional and structural residues in proteins Nucleic Acids Res 2006 34 Web Server W362 365 16845026 10.1093/nar/gkl124 PMC1538847

[B50] Nakai K Horton P PSORT: a program for detecting the sorting signals of proteins and predicting their subcellular localization Trends Biochem Sci 1999 24 34 35 10.1016/S0968-0004(98)01336-X 10087920

[B51] Cserzo M Wallin E Simon I von Heijne G Elofsson A Prediction of transmembrane alpha-helices in procariotic membrane proteins: the Dense Alignment Surface method Prot Eng 1997 10 673 676 10.1093/protein/10.6.673 9278280

[B52] Hirokawa T Boon-Chieng S Mitaku S SOSUI: classification and secondary structure prediction system for membrane proteins Bioinformatics 1998 14 378 379 10.1093/bioinformatics/14.4.378 9632836

[B53] Tusnády GE Simon I The HMMTOP transmembrane topology prediction server Bioinformatics 2001 17 849 850 10.1093/bioinformatics/17.9.849 11590105

[B54] Kelley LA MacCallum RM Sternberg MJE Enhanced genome annotation using structural profiles in the program 3D-PSSM J Mol Biol 2000 299 499 520 10.1006/jmbi.2000.3741 10860755

[B55] Larkin MA Blackshields G Brown NP Chenna R McGettigan PA McWilliam H Valentin F Wallace IM Wilm A Lopez R Thompson JD Gibson TJ Higgins DG Clustal W and Clustal X version 2.0 Bioinformatics 2007 23 2947 2948 10.1093/bioinformatics/btm404 17846036

[B56] Abascal F Zardoya R Posada D ProtTest: selection of best-fit models of protein evolution Bioinformatics 2005 21 2104 2105 10.1093/bioinformatics/bti263 15647292

[B57] Ronquist F Huelsenbeck JP MRBAYES 3: Bayesian phylogenetic inference under mixed models Bioinformatics 2003 19 1572 1574 10.1093/bioinformatics/btg180 12912839

[B58] Guindon S Gascuel O A simple, fast, and accurate algorithm to estimate large phylogenies by maximum likelihood Systematic Biology 2003 52 696 704 10.1080/10635150390235520 14530136

[B59] Stamatakis A Ludwig T Meier H RAxML-III: a fast program for maximum likelihood-based inference of large phylogenetic trees Bioinformatics 2005 21 456 463 10.1093/bioinformatics/bti191 15608047

