# Supplementary material for: Comparative analysis of plant genomes allows the definition of the "Phytolongins": a novel non-SNARE longin domain protein family
Source: BMC Genomics. 2009 Nov 4;10:510. doi: 10.1186/1471-2164-10-510 (PMC2779197; doi:10.1186/1471-2164-10-510)
Supplement: Additional file 2 — Table S2. Whole complements of "classic" longins from a number of model plant species. [file 1471-2164-10-510-S2.doc]

|  | **VAMP7** | | **Ykt6** | **Sec22** |
| --- | --- | --- | --- | --- |
|  | **71** | **72** |  |  |
| ***Populus trichocarpa*** | 5 | 8 | 2 | 3 |
| Arabidopsis thaliana | 4 | 7 | 2 | 2 |
| ***Oryza sativa*** | 3 | 6 | 2 | 4 |
| ***Physcomitrella patens*** | 3 | 5 | 2 | 2 |
| ***Volvox carteri*** | 1 | 3 | 1 | 1 |
| ***Chlamydomonas reinhardtii*** | 1 | 4 | 1 | 1 |
| ***Ostreococcus tauri*** | - | 1 | 1 | 1 |
