# Supplementary material for: Comparative analysis of plant genomes allows the definition of the "Phytolongins": a novel non-SNARE longin domain protein family
Source: BMC Genomics. 2009 Nov 4;10:510. doi: 10.1186/1471-2164-10-510 (PMC2779197; doi:10.1186/1471-2164-10-510)
Supplement: Additional file 3 — Alignment of the VAMP727 loop region in diverse plants. This figure shows a multiple alignment of the conserved VAMP727 acidic loop region including a few adjacent C-ter and N-ter residues, corresponding to regions 94-121 or 96-124 of VAMP727 proteins from respectively Arabidopsis thaliana or Oryza sativa. Loops regions are 98-116 (At) and 100-119 (Os) respectively. [file 1471-2164-10-510-S3.pdf]

**Gnetophyta**	
Gnetum gnemon	GRATETSG-AHNLG-----VDYGNDEKFKI
**Coniferophyta**	
Picea glauca	GGRAETNM-AHSL-----DKDYGYEEKFSV
Pinus taeda	GGRAETSM-AHSL-----DKDYGYEEKFSV
Picea sitchensis	GGRAETSM-AHSL-----DKDYGYEEKFSV
**Magnoliids**	
Saruma henry	GASITSES-PHPLADD--EDDYLLEDRFGI
**Monocotyledones**	
Phalaenopsis equestris	QSSINADG-SHPLIDD-DDDD-LFEDRFSI
Allium cepa	GSQINVD--PHPLADD-DDED-LFEDRFSI
Elaeis oleifera	GLGINDGG-FHTLADD-DDDD-LFEDRFSI
Ananas comosus	GSSIGSED-PHPLADE--EDDFLFEDRFSI
Oryza sativa	GSSIDEEG-QHPLADDADDDDFLLEDRFSI
Zea mays	GSSIDEEG-QHPLADDADEDDFLFEDRFSI
Triticum aestivum	GSSIDEEG-QHPLADDADEDDFLFEDRFSI
Triticum turgidum	GSSIDEEG-QHPLADDADEDDFLFEDRFSI
Hordeum vulgare	GSSIDEEG-QHPLADDADEDDFLFEDRFSI
Brachypodium distachyon	GSSIDEEG-QHPLADDADEDDFLFEDRFSI
Sorghum bicolor	GSSIDEEG-QHPLADDADEDDFLFEDRFSI
Saccharum officinarum	GSSIDEEG-QHPLADDADEDDFLFEDRFSI
Agrostis capillaris	GSSIDEEG-QHPLADDADEDDFLFEDRFSI
Zingiber officinale	GQSISGNN-SHPLIDE--EDADLFGDRFST
**Eudicotyledones**	
Aquilegia formosa	GASIGSEG-PHALADE--EDEDLFEDRFSI
Beta vulgaris	AGSIKSGD-AHPLAEENDSDDDLFEDRFSI
Vitis vinifera	GGSIRSDG-PHPLADEDDDDDDLFEDRFSI
Juglans hindsii x Juglans regia	SARIKDEG-PHPLADDEDEDDDLFEDRFSI
Juglans regia	GPSIKDVG-PHPLADDD-DDDDLFEDRFSI
Cucumis melo	GSSIKDEN-PHPLADDE-DDDDLFLDRFSI
Prunus persica	GSNNKIEG-PHPLADDN-EDDDLFEDRFSI
Fragaria vesca	APSIKNEG-PHPLADED-EDDALFEDRFSI
Malus x domestica x Malus sieversii	AADIKNAG-PHPLDDDD-EEDDLFEDRFSI
Malus x domestica	GPDIKNAG-PHPLADGD-EDDDLFEDRFSI
Glycine max	GASIKNDG-AHPLADDE-DDDDLFEDRFSI
Lotus japonicus	GASIKNAS-DHPLADDD-DDDDLFQDRFSI
Populus trichocarpa	SASIKNE--AHPLADDD-DDDDLFEDRFSV
Populus trichocarpa	GASIKNE--AHPLADDD-DDDDLFEDRFSI
Euphorbia esula	GSCISNE--VHPLADD--EDDDLFEDRFSI
Manihot esculenta	GASIKNE--THPLADD--DDDDLFEDRFSI
Brassica napus	EASIKNDE-PHPLADDD-EDDDLFGDRFSI
Brassica oleracea	GASIKIDE-PHSLADDD-EDDDLFGDRFSI
Descurainia sophia	EASIKNDE-RHPLADDD-EDDDLFGDRFSI
Arabidopsis thaliana	EASIKNDE-RHPLADED-EDDDLFGDRFSV
Brassica rapa	GASIKNDE-PHPLADDDDEDDDLFGDRFCI
Raphanus raphanistrum	EASIKNDE-PHPLADDDDEDDDLFGDRFSI
Raphanus sativus	EASIKNDE-PHPLADDDDEDDDLFGDRFSI
Gossypium raimondii	GASIRNEG-PHPLADDD-DDDDLFEDRFSI
Gossypium hirsutum	GASIRNGG-PHPLADDD-DDDDLFEDQFSI
Citrus clementina	GASIQNEE-SHPLADDD-EDDDLFEDRFSI
Vaccinium corymbosum	GASIRTDD-QHPLADDD-EDDDLFQDRFSI
Solanum habrochaites	GSSIKNDGDPHPLADDDEEDDDLFGDRFSI
Solanum tuberosum	GSSIKNDGDPHPLADDDEEDDDLFGDRFSI
Solanum lycopersicum	GSSIKNDGDPHPLADGDEEDDDLFGDRFSI
Solanum chacoense	GSSIKNDGDPHPLADDNEEDDDLFGDRFSI
Nicotiana tabacum	GSSIKNDGDPHPLADDEEEDDDLFGDRFSI
Ipomoea nil	GASIKSDG-PHPLADDDDEDDDLFEDRFSI
Triphysaria pusilla	GGSIKSDN-PHTLADDDDEDDDLFEDRFSI
Catharanthus roseus	GASIKPDG-PHPLADDDEEDDDLFEDRFGI
Coffea canephora	GASIKTDG-SHPLADDDDDDD-LFQDRFSI
Antirrhinum majus	GDSIKDDD-PHPLADDEDDD--LFEDRFSI
Lactuca saligna	GASIGND---HPLADDSDDD--LFEDRFSI
Lactuca perennis	GASIGND---HPLADDSDDD--LFEDRFSI
Lactuca serriola	GASIGND---HPLADDSDDD--LFEDRFSI
Lactuca sativa	GACIGND---HPLADDSDDD--LFEDRFSI
Centaurea maculosa	GASIGND---HPLADDSDDD--LFEDRFSI
Centaurea solstitialis	GASIGND---HPLADDSDDD--LFEDRFSI
Cichorium intybus	AASIGND---HPLADDSDDD--LFEDRFSI
Carthamus tinctorius	GASIGND---HPLADDSDDD--LFEDRFSI
Taraxacum kok-saghyz	GASIGND---HPLADDSDDD--LFEDRFSI
Helianthus paradoxus	GASIGSD---HPLADDSDDD--LFGDRFSI
Helianthus exilis	GASIGSD---HPLADDSDDD--LFEDRFSI
Helianthus annuus	GASIGSD---HPLADDSDDD--LFEDRFSI
Helianthus petiolaris	GGSIGSDND-NPLAENSDDD--LFEDRFSI
Helianthus tuberosus	GGSIGTDND-NPLAEDSDND--LFEDRFSI
