# Supplementary figures and images for: Comparative analysis of plant genomes allows the definition of the "Phytolongins": a novel non-SNARE longin domain protein family
Source: BMC Genomics. 2009 Nov 4;10:510. doi: 10.1186/1471-2164-10-510 (PMC2779197; doi:10.1186/1471-2164-10-510)

YKT6

PL

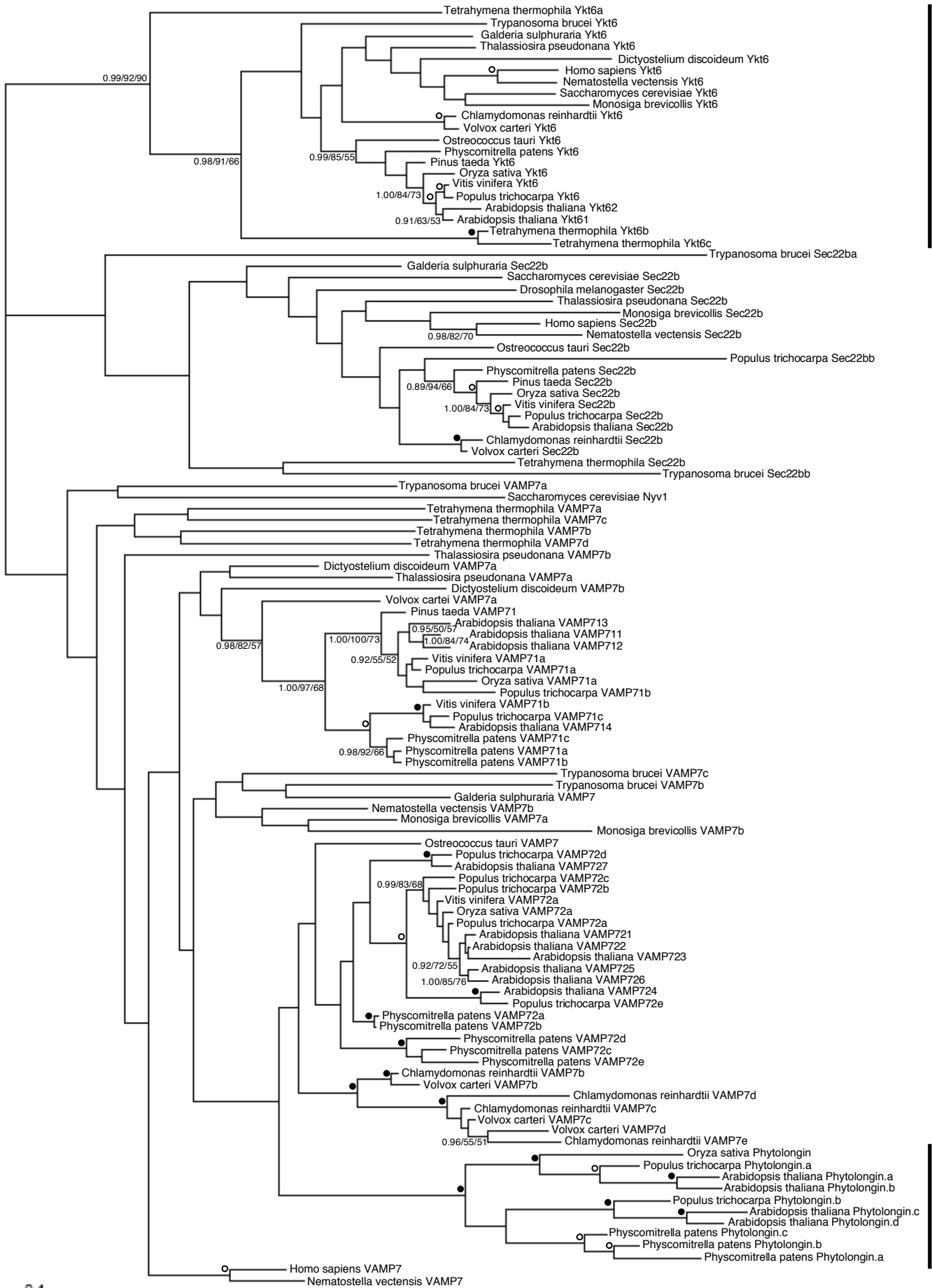

Supplement: Additional file 4 — Longin phylogeny. This figure demonstrates that the Phytolongins form a well-resolved clade within the longin family and that they are unlikely to have been derived from within the Ykt6 clade of longins. The vertical bars highlight the Ykt6 and Phytolongin (PL) clades respectively. [file 1471-2164-10-510-S4.pdf]

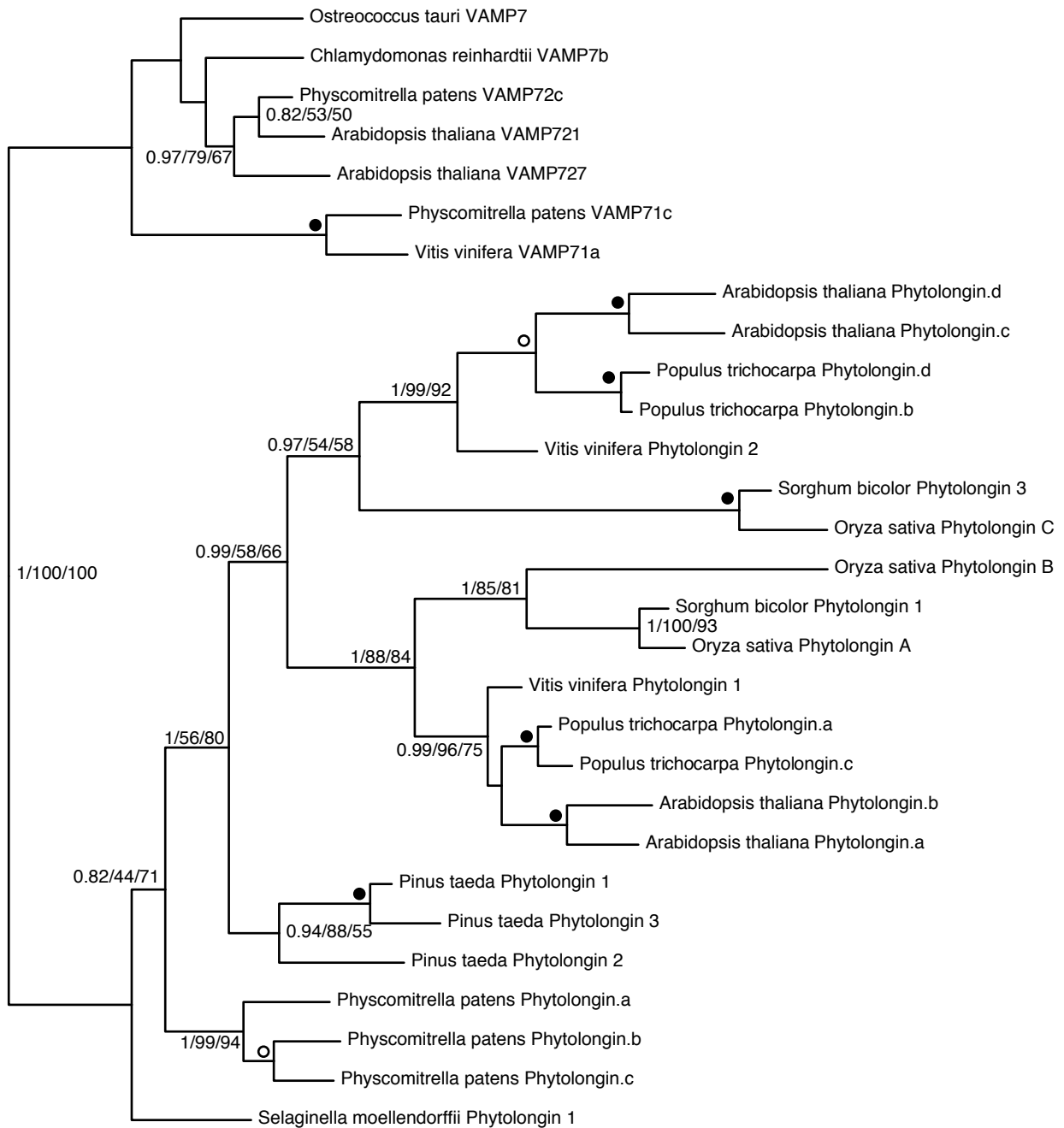

0.4

Supplement: Additional file 5 — Phytolongin phylogeny (altered taxon sampling). This figure shows the robust separation of bryophyte and gymno/angiosperm sequences. This dataset included a homologue from Salaginella moellendorffii but excluded a divergent sequence from Sorghum bicolor and resulted in a more robust resolution of the Physcomitrella patens sequences from the other plant Phytolongins (vertical bar). [file 1471-2164-10-510-S5.pdf]
